# Supplementary figures and images for: Retrospective screening of serum IgG glycosylation biomarker for primary Sjögren’s syndrome using lectin microarray
Source: PeerJ. 2023 Feb 22;11:e14853. doi: 10.7717/peerj.14853 (PMC9961092; doi:10.7717/peerj.14853)

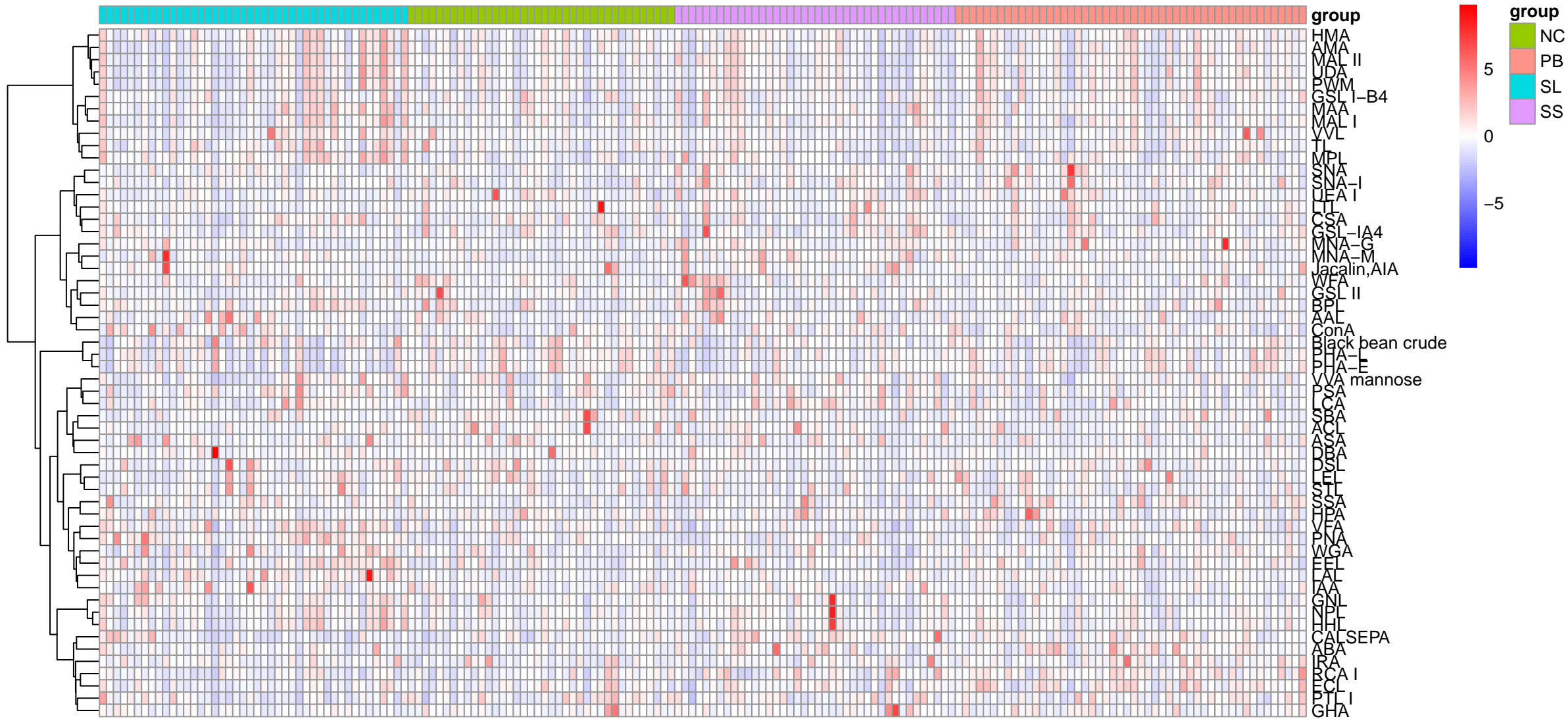

Supplement: Supplemental Information 1 — Rows: samples; columns: lectins. NC: healthy controls; PB: PBC patients; SL: SLE patients; SS: PSS patients. Results of SLE patient did not enter subsequential analysis. Color key indicates standardized fluorescent intensity for lectins: blue: lowest; red: highest. The heatmap was generated using R software (Version 4.0.2). [file peerj-11-14853-s001.pdf]

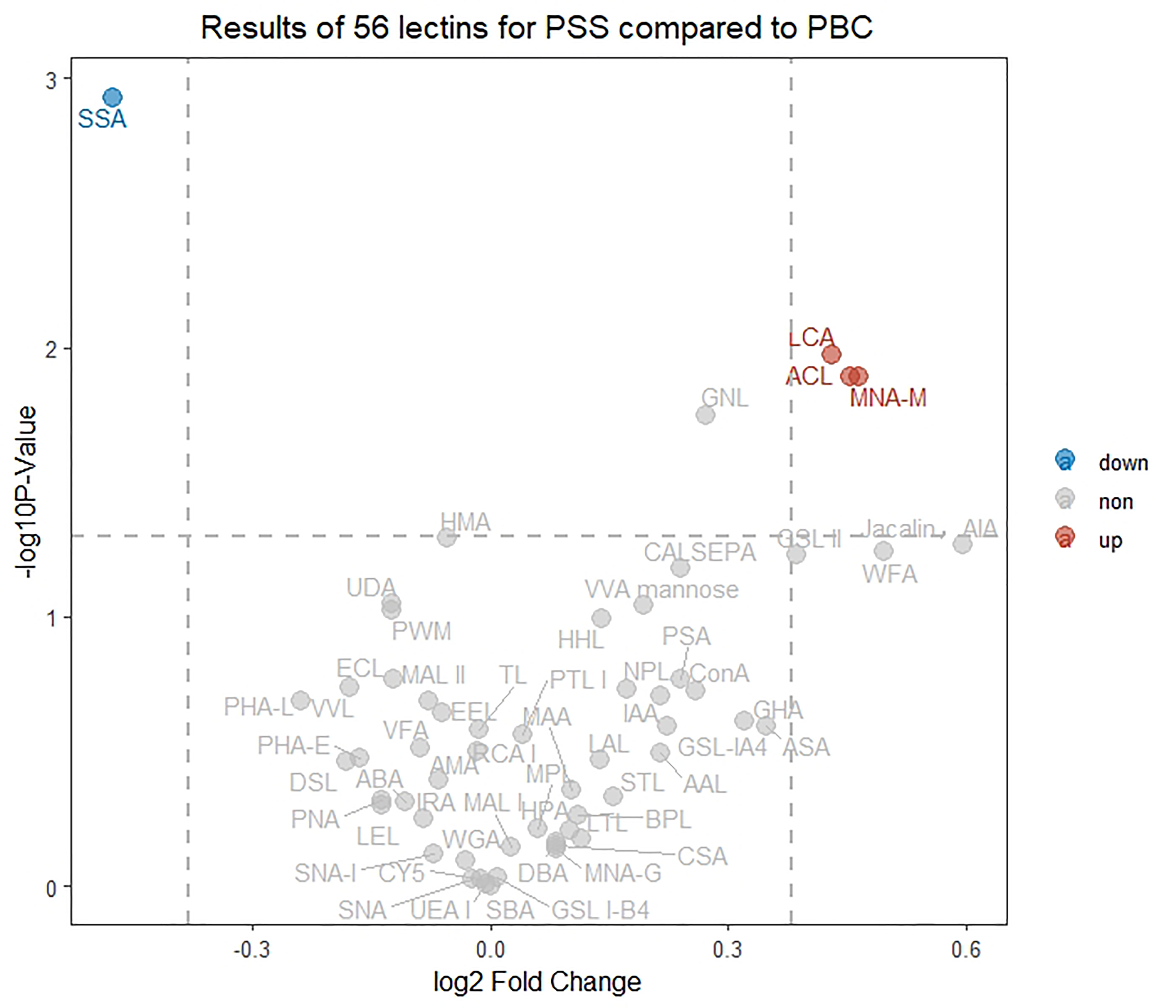

Supplement: Supplemental Information 2 — Volcano plot showing the log2 (fold change) of standardized fluorescent intensity and the p-value for the 56 lectins results. The red dots represent increased level of lectin (p < 0.05 and fold change fold change (group1 (S/N)/group2 (S/N)) ≥1.3), whereas blue dots represent decreased level of lectin (p < 0.05 and fold change fold change (group1 (S/N)/group2 (S/N)) <0.77). The volcano plot was generated using R software (version 4.0.2). [file peerj-11-14853-s002.tif]

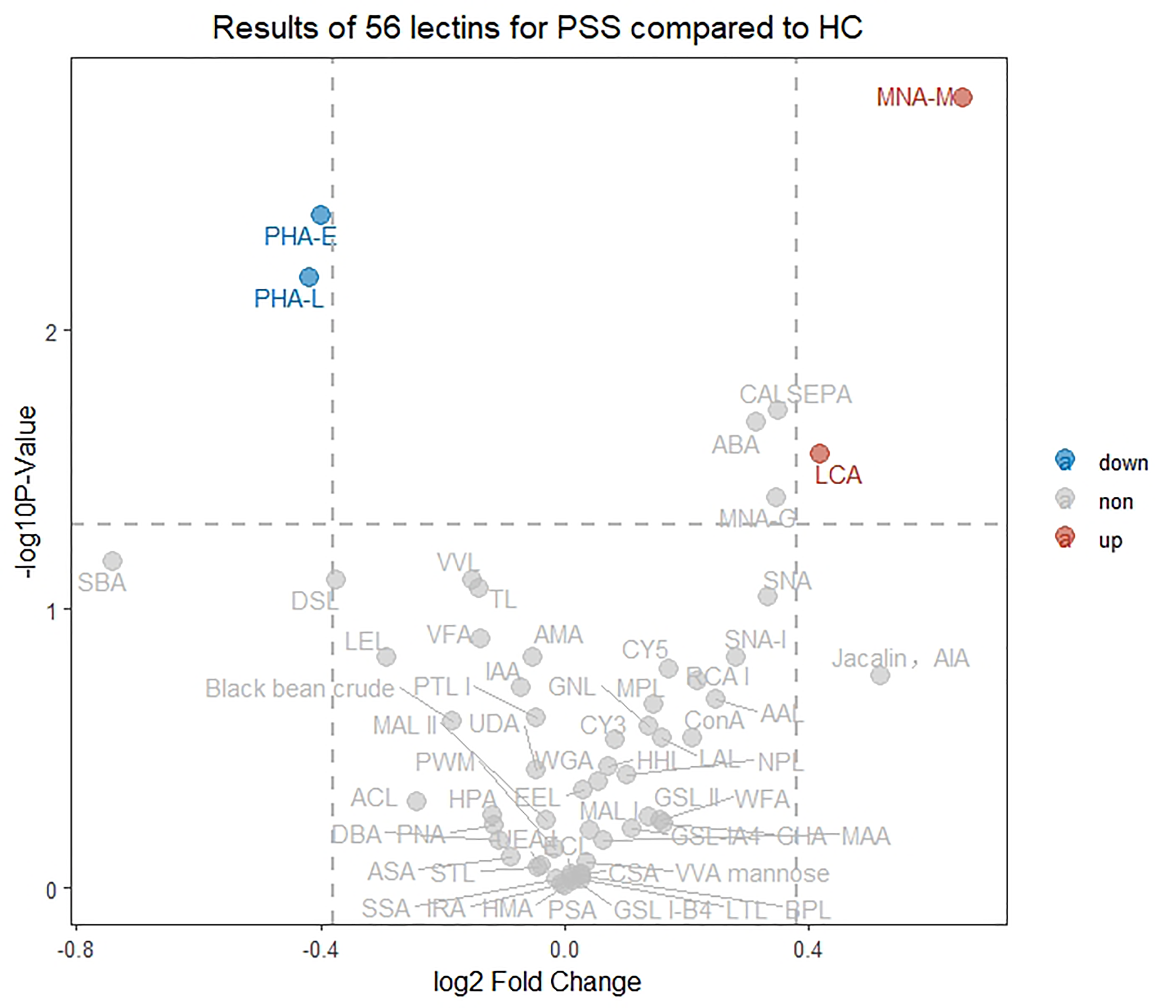

Supplement: Supplemental Information 3 — Volcano plot showing the log2 (fold change) of standardized fluorescent intensity and the p-value for the 56 lectins results. The red dots represent increased level of lectin (p < 0.05 and fold change fold change (group1 (S/N)/group2 (S/N)) ≥1.3), whereas blue dots represent decreased level of lectin (p < 0.05 and fold change fold change (group1 (S/N)/group2 (S/N)) <0.77). The volcano plot was generated using R software (version 4.0.2). [file peerj-11-14853-s003.tif]
