# Supplementary material for: Retrospective screening of serum IgG glycosylation biomarker for primary Sjögren’s syndrome using lectin microarray
Source: PeerJ. 2023 Feb 22;11:e14853. doi: 10.7717/peerj.14853 (PMC9961092; doi:10.7717/peerj.14853)
Supplement: Supplemental Information 4 [file peerj-11-14853-s004.docx]

Miame Checklist ***Part 1 Experiment description***

A total of 128 serum samples were used for lectin microarray analysis, obtained from 40 PSS patients, 50 PBC patients, and 38 healthy controls who were healthy volunteers without autoimmune diseases. In addition, a new cohort of samples was collected to verify significant findings using lectin blot, including 16 PSS patients, 16 PBC patients, and 16 healthy controls.

***Part 2 Array design***

Totally 128 serum samples were detected using a commercial lectin microarray (BCBIO Biotech, Guangzhou, China) with 56 lectins.

***Part 3 Samples***

Serum samples were obtained by separation from peripheral blood and stored at -80 °C until use. Autoantibodies were tested using chemiluminescence immunoassay.

***Part 4 Hybridizations***

lectin microarrays were taken out from −80 °C and warmed up at room temperature for half an hour, then they were incubated with a blocking buffer (3% BSA in PBS) at room temperature for 2h. After washing three times with PBS, 200μl of 1:1000 diluted samples serum were added and incubated with the microarrays at 4 °C overnight. The microarrays were washed three times with PBS and then incubated with 5mL of 1:1000 diluted Cy5-labeled goat anti-human IgG antibody (Jackson Laboratory, Bar Harbor, ME) in the dark at room temperature for 50min. Finally, after three times PBS washes, microarrays were rinsed with D.I. water and dried.

***Part 5 Measurements***

Microarrays were scanned with the GenePix 4000B Microarray Scanner (Molecular Devices, Sunnyvale, CA). For lectin array assays, the median foreground and background fluorescent intensity for each spot on the arrays were acquired using GenePix Pro 6.0 software. We calculated the signal-to-noise ratio (S/N) (the medium intensity of the spot foreground relative to the background) of each lectin spot. The following rules were used to identify significant differences in the binding activity of lectins between subject groups: (a) fold change[group1(S/N) / group2(S/N)] ≥1.3 or <0.77, (b) P-value <0.05.

***Part 6 Normalization controls***

To prevent bias of the lectin microarray from the inter-array, we normalized the S/N data in terms of controls between arrays.
